# Electronic Nose Humidity Compensation System Based on Rapid Detection

**DOI:** 10.3390/s24185881

**Published:** 2024-09-10

**Authors:** Minhao Cai, Sai Xu, Xingxing Zhou, Huazhong Lu

**Affiliations:** 1 College of Engineering, South China Agricultural University, Guangzhou 510642, China; caiminhao@stu.scau.edu.cn; 2 Institute of Facility Agriculture, Guangdong Academy of Agricultural Sciences, Guangzhou 510640, China; zhouxingxing@gdaas.cn; 3 Guangdong Academy of Agricultural Sciences, Guangzhou 510640, China; huazlu@scau.edu.cn

**Keywords:** electronic nose, humidity compensation, random forest, rapid detection

## Abstract

In this study, we present an electronic nose (e-nose) humidity compensation system based on rapid detection to solve the issue of humidity drift’s potential negative impact on the performance of electronic noses. First, we chose the first ten seconds of non-steady state (rapid detection mode) sensor data as the dataset, rather than waiting for the electronic nose to stabilize during the detection process. This was carried out in the hope of improving the detection efficiency of the e-nose and to demonstrate that the e-nose can collect gasses efficiently in rapid detection mode. The random forest approach is then used to optimize and reduce the dataset’s dimensionality, filtering critical features and improving the electronic nose’s classification capacity. Finally, this study builds an electronic nose humidity compensation system to compensate for the datasets generated via rapid real-time detection, efficiently correcting the deviation of the sensor response caused by humidity variations. This method enhanced the average resolution of the electronic nose in this trial from 87.7% to 99.3%, a 12.4% improvement, demonstrating the efficacy of the humidity compensation system based on rapid detection for the electronic nose. This strategy not only improves the electronic nose’s anti-drift and classification capabilities but also extends its service life, presenting a new solution for the electronic nose in practical detecting applications.

## 1. Introduction

**Figure 1:**
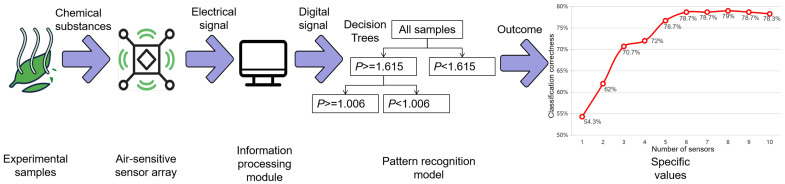
Signal detection flow.

An electronic nose is a device that duplicates the human olfactory system, with the notion being presented in the 1960s [1]. It stimulates the olfactory process by establishing a potential difference by interacting with several kinds of gas-sensitive sensors and gas particles. With the advancement of technology, electronic noses have become widely used in a variety of industries, including food and agricultural product detection [2,3,4,5], biological detection [6,7], medical diagnostics [8,9,10,11], environmental monitoring [12,13,14], and others, demonstrating their broad application potential and significant value. The electronic nose system consists of two major components: a gas-sensitive sensor array and a pattern recognition model. The gas-sensitive sensor array collects and transforms gas data, while the pattern recognition model analyzes, predicts, and recognizes them. Figure 1 illustrates the specific flow of signal detection. The advantages of an electronic nose are a rapid identification capability, a high-precision recognition performance, ease of use, and portability. Metal oxide semiconductor (MOS) sensors are the most popular gas sensor array modules, due to their cheap price and long operational life. This sort of sensor not only ensures the electronic nose’s efficiency but also offers apparent advantages in terms of commercial application and process maturity, making it the most comprehensive and mature sensor type on the market today.

However, drift frequently impairs the performance stability of electronic noses [15], making it impossible to verify their stability in specific tasks. These drift factors include environmental humidity, ambient temperature, and “olfactory fatigue” caused by long working hours, among others, which can cause deviations in the sensor’s response and a change in the baseline, affecting the accuracy and reliability of the prediction model, as well as decreasing the performance and service life of the electronic nose. Frequent sensor replacement not only increases detection costs but also necessitates recalibration and the development of a new identification model to react to the baseline shift caused by differences in sensor-manufacturing processes. Data compensation using mathematical modeling is an efficient and cost-effective approach to take. This method attempts to algorithmically adapt the gathered data in order to reduce the negative impacts of drift, lengthen the working life of the electronic nose, and increase its stability. However, the drift problem is complicated since it impacts the response signals of all of the electronic nose’s sensors, and the drift mechanisms and patterns are difficult to characterize specifically using mathematical models. The unpredictability, uncertainty, and dynamics of drift make it difficult to compensate electronic noses accurately. These limit the efficacy of electronic noses in certain application settings. Thus, strengthening the flexibility and robustness of electronic noses to drift effects via drift compensation algorithms is critical to increasing their efficiency in certain application circumstances. This necessitates not only the modification and optimization of the existing mathematical model but also an in-depth examination of the attempts of the detection mechanism of the electronic nose itself, in order to increase the electronic nose’s operational efficiency. Since compensation itself is difficult to express using direct linear mathematical formulas or data, the compensation effect of an electronic nose is often determined by looking at the data distribution of a principal component analysis plot or the classification results of a classification mathematical model.

Among the electronic nose drift factors, the drift problem caused by changes in environmental humidity is an important factor affecting its stability and accuracy [16]. Liao Youwei et al. [17] proposed a back propagation neural network combined with a genetic algorithm to compensate for the problem that isopropanol gas sensors are susceptible to changes in ambient humidity, which leads to a decrease in accuracy so that the stability of the electronic nose in the detection has been improved. To solve the problem of data distortion caused by the drift of the electronic nose, Sun Fangyu et al. [18] compensated for the drift of the electronic nose using the method of cross-domain active learning and, when compared, the different periods were able to achieve better accuracy. As far as we know, fewer attempts have been made to collect or compensate the data when the gas data collected by the e-nose are undergoing preliminary differentiation (i.e., the fast detection mode proposed in this paper). Therefore, this paper proposes a humidity drift compensation model for the electronic nose based on fast detection to improve the detection performance and service life of the electronic nose, as well as to solve the drift problem caused by environmental humidity. The compensation attempt mainly involves the following aspects:

Humidity compensation in fast detection mode: the electronic nose’s fast classification effect is achieved by shortening the detection time and leveraging the fast reaction of different sensors when the initial discrepancy occurs. This method not only minimizes the time of each detection of the electronic nose, but it also increases its operating efficiency and has a good categorization effect.

Feature optimization and data dimensionality reduction: The random forest method is used to improve the features and lower the dimensionality of the electronic nose data collection. This method effectively avoids the overfitting problem caused by dimension explosion, while keeping the classification effect of the electronic nose and decreasing the complexity of the compensation model.

Establishment of humidity compensation model: An electronic nose humidity compensation model is built, and the model is trained using the source domain, i.e., the corrected dataset, to accomplish convergence and prediction of drifting data. This model increases e-nose correction, anti-drift performance, and detection stability.

The results of this study show that rapid detection, when the sensor initially detects concentration differences, combined with the development of a humidity compensation model to compensate the data, can significantly improve the recognition effect and efficiency of the electronic nose for specific gasses (for example, tea gas). The increased average resolution of the recognition system demonstrates the practicality of the methodology and provides a new direction for the detection method and drift compensation of the electronic nose.

## 2. Materials and Methods

### 2.1. Experimental Materials

In this study, five tea samples (Huangshan Maofeng, Yinghong No. 9, Xihu Brand, Biluo Brand, and Xinyang Maojian) were selected to collect data to completely evaluate the electronic nose’s detecting capabilities under varied relative humidity conditions. Before the experiment began, each tea sample was carefully weighed at 1 g using an electronic scale and placed in a 100 mL beaker that had been pre-washed and naturally air-dried. To ensure that the experimental conditions were consistent, all beakers were prepared in an unscented, shady environment with no external disturbance. During the experiment, we kept the tea samples at 25 °C and varied the relative humidity gradient to 35%, 50%, 65%, 80%, and 95%. Each time tea gas data were collected, a single sample was first removed from the constant temperature and humidity chamber. It was then collected by the electronic nose until the end of the electronic nose’s gas-sensing session and then the next sample was taken out to repeat the experiment, thus obtaining all the data. Relative humidity, an important environmental factor influencing electronic nose operation, can affect sensor sensitivity and baseline drift. Relative humidity is defined as the ratio of the partial pressure of water vapor in air to the saturated vapor pressure of water at the same temperature and is commonly stated as a percentage, while absolute humidity defines the amount of water per unit volume of air. However, relative humidity was chosen for this study since it is better suited to the e-nose’s ambient circumstances in actual applications and testing. Tea samples were allowed to adjust to their surroundings in the test chamber for 30 min at each humidity level before being removed and covered with a double layer of cling film. Data were collected using the headspace sampling technique.

### 2.2. Experimental Equipment

**Figure 2:**
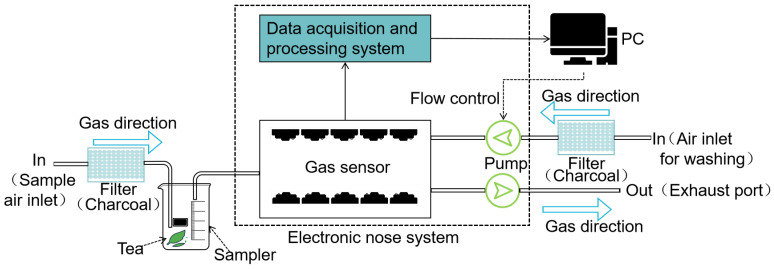
Electronic nose data acquisition schematic.

We collected gas concentration data using the PEN3.5 electronic nose system (provided by Airsense Analytics GmbH company, Schwerin, Germany), which is frequently used in laboratories. Table 1 [19] shows the sensitive gas categories corresponding to the different types of sensors of the PEN3.5 electronic nose. The system consists of four basic components: a gas pump system, a control module, a gas sensor module, and a signal-data-processing and storage module. The gas sensor, as the electronic nose’s main component, generates potential changes through redox reactions with specific gas particles, whilst the signal-data-processing and storage module turns the sensor’s electrical signals into quantifiable gas concentration data. These sensors can detect and respond to a variety of volatile organic compounds in tea gas evaluation and discrimination, providing a sound scientific foundation for analyzing tea quality. The PEN3.5 electronic nose system employs ten different types of gas-sensitive sensors, each with a high level of sensitivity and selectivity for a specific class of gasses, enabling the precise evaluation and categorization of gaseous molecules. The DHTHM temperature and humidity chamber (supplied by DHT&reg company, model DHTHM-27-O-P-ES) was utilized to control the temperature and humidity of the experiment. Figure 2 shows the data acquisition schematic of the electronic nose.

### 2.3. Dataset Preparation and Experimental Methods

To obtain the dataset required for the experimental analysis, sampling operations were performed on each tea sample in various humidity settings, as shown in Table 2 below. The particular parameter settings for the experiment were as follows: the electronic nose sampling cleaning time was set to 60 s, the interval for collecting gas data was 1 s, the time spent waiting for the sensor to zero out was 10 s, the sample inlet time was 5 s, the time spent analyzing the sampling data was 60 s, the gas flow rate was 190 mL/min, and the time spent determining the concentration of the collected gas was 60 s. We used a computer to control the flow rate parameters of the electronic nose to ensure the consistent sampling of all samples.

**Figure 3:**
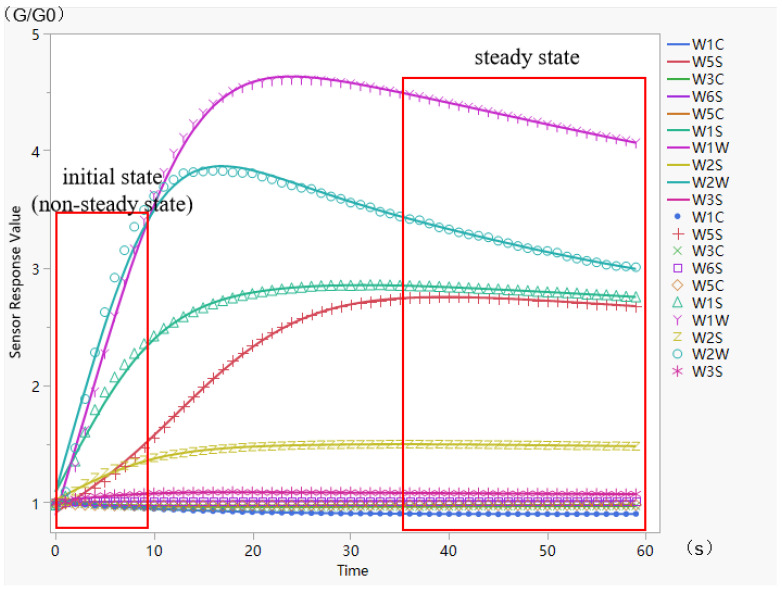
Electronic nose’s gas concentration time profile.

Figure 3 shows the gas concentration graph over time for the electronic nose during the detection procedure (the horizontal coordinates of the graph are seconds and the axial coordinates are the response values of the electronic nose). In routine electronic nose detection, the value when the gas enters a steady state is commonly utilized for classification analysis [20] to verify that the classification results are correct. However, the graph demonstrates that, 10 s after the start of the sample’s electronic nose detection, the reaction of the various sensors to the gas clearly stratified and differed. In this study, an innovative method is provided to perform rapid detection when the gas concentration has not yet entirely stabilized and to use the dataset at this point for compensatory analysis, with the goal of enhancing the detection efficiency and classification results of the electronic nose. The individual datasets are classified as follows: first, the average value of gas concentration before ten seconds, and second, the value with the greatest deviation from the beginning concentration. This experiment used fewer data dimensions to examine the detection of the beginning state, which is because we hope to achieve electronic nose detection with fewer dimensions and to lower the complexity of the data-processing model to assure its generality. Furthermore, because we need to compare to the steady state, and the meaning of the different dimension values in the smooth state is more similar, we reduced the dimensionality of the data to assure the credibility of the comparison.

As far as we know, fewer attempts have been made to collect datasets in the initial state of electronic nose concentration collection for analysis, and efforts in this direction can help to not only shorten detection time, improve the efficiency of electronic noses, and extend their service life, but also to enhance the anti-drift capability of electronic noses through compensation and analysis.

### 2.4. Data-Processing Methods

#### 2.4.1. Classification Modeling System

In our study, we used the following three popular machine learning algorithms: support vector machine (SVM), whose strength is its ability to search in high-dimensional areas and optimize inter-class borders, resulting in improved classification accuracy; K-nearest neighbor (KNN), which classifies unknown samples based on their distance from a known sample, often using the majority voting criterion; and artificial neural networks (ANNs), which use nonlinear mapping to imitate the network structure of neurons in the human brain and perform complex pattern recognition tasks. Various algorithms are used to create a comprehensive classification system that analyzes and classifies the e-nose’s gas detection data, with the purpose of assessing the e-nose’s classifications and the stability of its compensatory system. Table 3 shows the parameters of each mathematical model.

The formula for the rate of correct classification of these is as follows:

(1) $$Accuracy=\frac{Number\;of\;Correctly\;Classified\;Instances}{Total\;Number\;of\;Instances}$$

The formula for the average correctness of these is as follows:

(2) $$y=\frac{\sum_{i=1}^{n}x_{n}}{n}$$

where y is the average rate of correct classification, n is the number of classification models, and xn is the classification correctness rate of the corresponding classification model.

#### 2.4.2. Feature Optimization and Compensation Systems

In this experiment, random forest (RF) [21] was employed to optimize features and compensate for electronic nose drift. RF is a parallel type of integrated learning method that uses a decision tree to generate numerous independent classifiers, resulting in a forest structure. This form of integrated learning method may successfully avoid the problem of unstable results caused by a single feature selection technique, as well as optimize the results and eliminate potential overfitting issues by utilizing a large number of decision tree models. Wijaya et al. [22] have demonstrated the potential of the random forest method in electronic nose signal processing in their study. Their article demonstrates that the integrated learning class algorithm is more accurate than the typical single classifier, that it can avoid the influence of a few samples on the overall compensation due to large bias, and that it improves the stability of the compensation model; the advantages mentioned above ultimately prompted us to choose random forest as our compensation model.

**Figure 4:**
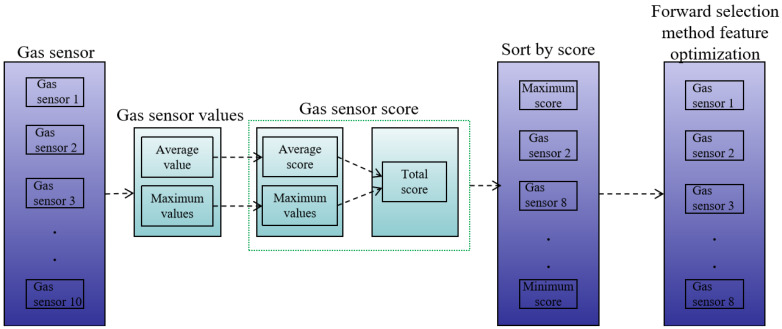
Flow chart of feature optimization.

In this work, two crucial eigenvalues were chosen: the average gas concentration 10 s ago and the value with the greatest deviation from the initial concentration. The feature matrices were built with dimensions of 10 (number of sensors) × 2 (eigenvalues) × 5 (percentage humidity), totaling 100 dimensions, and their RF classification scores were calculated. By adding the scores of these two features, we calculated the overall feature prioritization score for each sensor, which reflects the sensors’ combined relevance in the classification process. Finally, the sensor scores were weighted and inserted into the classification model’s output findings to achieve the best sensor combination. This allowed us to assess the value of each gas-sensitive sensor in the classification process, as well as to choose and optimize features for the sensor combination. Figure 4 depicts a specific feature optimization flowchart.

By selecting samples and features from the original dataset, the impact of individual samples on overall classifier performance is decreased, resulting in improved classification accuracy and robustness. At the same time, lowering data dimensionality can help to avoid the “dimensionality explosion” problem, while also enhancing categorization and compensation for electronic nose detection.

This experiment also used the RF algorithm to create an electronic nose humidity compensation model, which predicts and converges drift data using data prediction, to improve the electronic nose’s stability and accuracy in varying humidity settings. In order to evaluate the effectiveness of the e-nose, we take the accuracy of tea classification as an evaluation metric and divide all the samples into a training set and a test set in a ratio of four to one.. And, through repeated experimental tests, we determined the model’s key parameters: the number of forest trees was set to 100, the number of extraction terms in splitting was 17, and random sampling was performed using the Bootstrap method, which not only ensured sample representativeness but also strengthened the model’s generalization ability.

## 3. Results and Discussions

### 3.1. Effect of Ambient Humidity on the Classification Results of Electronic Nose

Ambient humidity is a typical drift factor that affects the performance of electronic noses and the sensitivity of gas sensors. Yan and other researchers [23] found that, as relative humidity increases, the sensors’ responsiveness decreases. However, in low humidity situations, the e-nose’s sensitivity increases substantially, potentially amplifying environmental disturbances and affecting the e-nose’s classification function.

In this experiment, we investigated the resolution of tea under various relative humidity settings in the presence of a constant gas concentration, and the experimental results are presented in Table 4. The results reveal that average resolution gradually increases as relative humidity increases, which contradicts prior studies’ findings. This mismatch could be due to the fact that humidity influences not only the performance of the gas sensor but also the concentration of gasses emitted from agricultural goods, which indirectly impacts the electronic nose’s detecting capability. Furthermore, the amount and types of volatile chemicals emitted by different types of agricultural products under varied humidity circumstances may vary, necessitating analysis and testing to acquire accurate analytical results in specific applications and research.

Because the influence of humidity change on the electronic nose is not powerful, and because there is no clear linear relationship, the mechanism is complex and difficult to define with a single mathematical function. Through machine learning modeling, we can more effectively capture and correct the sensor response deviation caused by humidity variations, thus enhancing the stability and accuracy of the electronic nose under various environmental conditions.

### 3.2. Comparison of Steady State and Rapid Test Results

In this study, we used a unique rapid identification compensation approach that selects the average value ten seconds ago and the maximum value of difference from the start as feature information in order to improve the detection efficiency and classification impact of the electronic nose. This rapid detection technique not only minimizes the average working time of the electronic nose but also helps to extend its service life and mitigates the detrimental influence that long working times can have on detection accuracy. However, we did not strictly define fast detection because we assumed that the initial differentiation time of the gasses detected by each product would differ, so we only chose a fixed ten seconds (where most sensors were able to peak in this experiment) to compare with a steady state to determine the possibility of fast detection.

Table 5 shows the discrimination accuracy of tea samples under rapid identification conditions. By comparing the average resolution of tea leaves under different relative humidity conditions, we discovered that, when using the initial state values for classification, the average correct classification rate of tea leaves outperformed the steady state for all four humidity gradients except 95% relative humidity, with up to 12% improvement. This phenomenon can be attributed to the fact that the difference between the mean and maximum difference is smaller in the steady state, which is equivalent to using only one feature for classification, thereby reducing the number of feature dimensions available for classification; the resulting reduction in the amount of information may lead to weak classification results.

The experimental results demonstrate that it is perfectly viable to use data from gas-sensitive sensors during early concentration differences (initial state) for rapid detection, compensation, and classification. This method can capture critical gas concentration information in a short period, and we intend to improve the classification accuracy of the electronic nose using effective compensation algorithms to ensure that the electronic nose’s anti-drift performance remains stable even under rapid detection conditions.

### 3.3. Comparison before and after Feature Optimization

The Pen3.5 electronic nose’s original sensor array included ten different sensors, each of which provided two feature values. The data used for feature optimization were experimentally acquired tea gas data for all humidity conditions. Following the feature optimization process of the RF algorithm, which is a forward selection approach, we were able to reduce the number of sensors to eight: W1S, W5S, W2S, W3S, W2W, W1W, W1C, and W5C. This optimization method reduced the number of sensors, resulting in fewer feature dimensions in the dataset and a simpler model.

**Figure 5:**
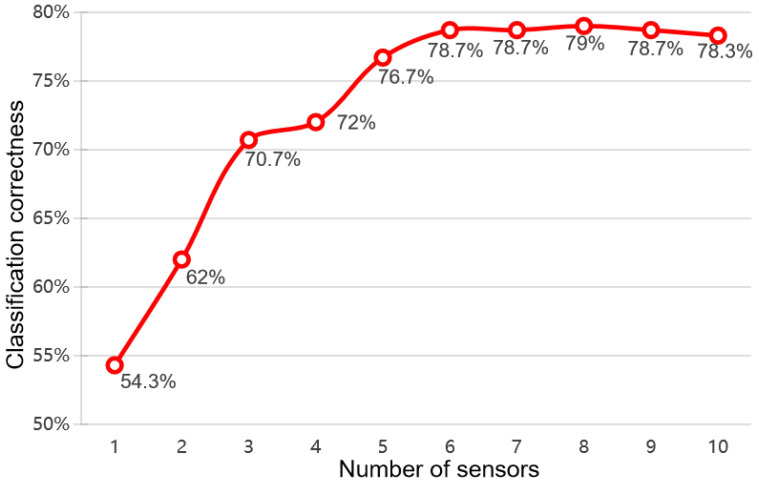
Feature optimization results.

Figure 5 shows the feature optimization results. In order to find the best feature optimization group, the datasets collected under all humidity conditions were used to participate in the feature optimization results, and the final optimization results were compared using the average correct discrimination rate of tea leaves. As can be seen from the results, the accuracy of the feature-optimized test set peaked at the streamlined combination of eight sensors, with an optimal discrimination correct rate of 79%. The optimization process was successful in significantly reducing the number of sensors and the dimensionality of the feature matrix with almost no impact on classification accuracy, which not only improves data-processing efficiency while ensuring that the effective information of the data is not lost, but also lowers the computational cost of the model.

### 3.4. Comparison of Results before and after Compensation

**Figure 6:**
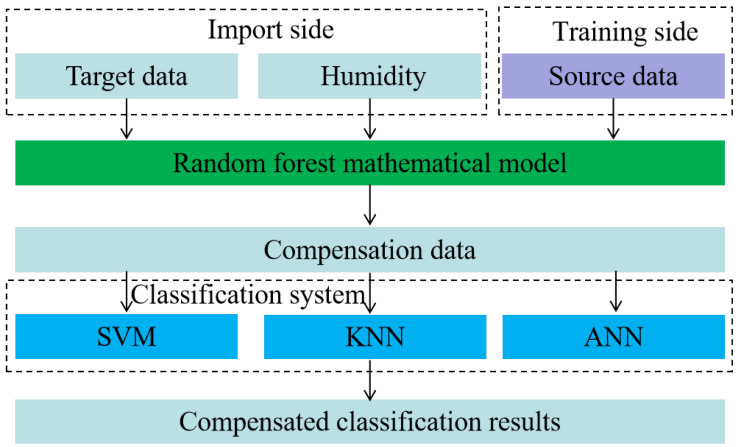
Flow chart of random forest compensation.

In this study, we performed an in-depth compensation analysis of the feature-optimized dataset using the RF compensation model, intending to improve the electronic nose’s classification accuracy and drift resistance. We chose to train in high relative humidity conditions (80% and 95%). This is because the electronic nose gave the best classification results under these conditions, demonstrating that the dataset is less prone to drift and has higher classification accuracy at these humidity levels. The datasets collected under these conditions were used as the source domain, or training dataset, to train the electronic nose’s humidity compensation model. Furthermore, we used the compensation model to handle the low humidity datasets (35%, 50%, and 65%), which have lower average classification results. Figure 6 shows the compensation process in detail.

Table 6 details the comparison results before and after compensation, displaying the average resolution after compensation for three humidity conditions: 35%, 50%, and 65%. The data revealed that the compensated training set average improved by 5.3%, from 92.7% to 98%. In comparison, the test set’s average resolution increased by 12.4%, from 87.7% to 99.3%. This large improvement demonstrates the compensation model’s usefulness while also significantly improving the electronic nose’s resilience and anti-drift capabilities, resulting in a good compensation impact.

**Figure 7:**
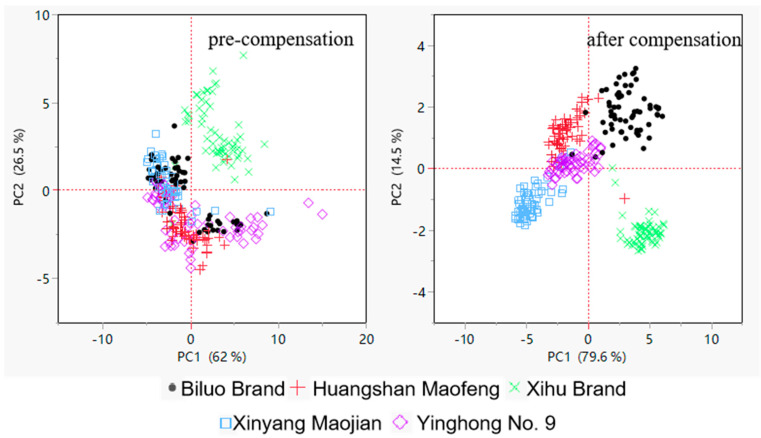
Comparison plot of principal component analysis before and after rapid test compensation.

Figure 7 depicts a comparison of principal component analysis (PCA) before and after adjustment under rapid testing settings. Before the correction, the distributions of different tea types (represented by different colors and shapes in the picture) had more intersections, and certain tea varieties’ sample points were relatively dispersed. This distribution characteristic suggests that the original data were not adequately separated between tea kinds. After the compensation model corrected for data drift, the corrected tea data had sharper cluster boundaries in the PCA graphic. This adjustment increased the distinction between tea kinds and clarified their confidence intervals in the PCA space. This increased clustering impact implies that the compensated model may effectively correct data drift while also increasing the model’s sensitivity for tea variety identification. As a result, the compensated PCA not only demonstrated the distinctness of tea kinds but also provided significant data support for the speedy and precise identification of electronic noses. This demonstrates the compensation model’s effectiveness and applicability in tea variety identification.

## 4. Conclusions

In this study, we proposed an electronic nose humidity compensation system based on rapid detection, to mitigate the impact of humidity drift on the electronic nose’s performance and increase classification accuracy while delaying sensor replacement and lowering overall detection costs. We demonstrated the rationale and benefits of the rapid detection method by comparing tea categorization results under fast detection and steady state. We then utilized the random forest model to optimize and adjust the dataset’s features gathered by the electronic nose. Finally, after optimizing and compensating the random forest mathematical model, the tea classification results improved by at least 12% on average, with test and training set results reaching 98% and 99.3%, respectively, and the electronic nose’s anti-drift performance improved significantly. Of course, this experiment only uses tea gas detection to demonstrate the feasibility of this approach. Still, it can point the electronic nose in a new direction for improving performance and compensation in practical applications such as agricultural product detection, environmental detection, etc.

## Figures and Tables

**Table 1:** Electronic nose gas sensors corresponding to different types.

Sensor Types	Sensitive Chemicals
W1C	aromatic compound
W5S	nitrogen oxide
W3C	aromatic ingredients, ammonia-based
W6S	mainly hydrides
W5C	aliphatic fragrance compounds
W1S	methane
W1W	organic sulfides and terpenes
W2S	mainly ethanol
W2W	aromatic components and sulfides
W3S	alkanes

**Table 2:** Number of samples of various types of tea under different humidity condition.

Humidity Level	35%	50%	65%	80%	95%
Huangshan Maofeng	20	20	20	20	19
Yinghong No. 9	20	20	20	20	20
Xihu Brand	20	20	20	20	20
Biluo Brand	20	20	19	20	20
Xinyang Maojian	20	20	20	20	20

**Table 3:** Parameters of the classification algorithm.

Learning Algorithms	Grid Search Parameters
SVM	Regularization parameter (C) = 1 Gamma = 0.1 Kernel = radial basis function (RBF)
KNN	n_neighbors (K) = 10 Weighting function=uniform
ANN	Layers = 1 Neurons = 3 Activation function = Sigmoid Learning rate = 0.1

**Table 4:** Smooth state of correct tea categorization.

Relative Humidity	SVM	KNN	ANN	Average Rate of Correct Classification
35%	89%/76%	71%/76%	93%/88%	84.3%/80%
50%	83%/88%	55%/72%	95%/84%	77.7%/81.3%
65%	92%/88%	76%/80%	89%/84%	85.7%/84%
80%	93%/92%	77%/84%	100%/100%	90%/92%
95%	99%/100%	97%/100%	100%/100%	98.6%/100%

(The training set is on the left side of the ‘/’ symbol and the test set is on the right side of the ‘/’ symbol).

**Table 5:** Correct classification of tea under rapid testing conditions.

Relative Humidity	SVM	KNN	ANN	Average Rate of Correct Classification
35%	95%/88%	88%/88%	100%/88%	94.3%/88%
50%	92%/84%	87%/92%	100%/92%	93%/89.3%
65%	99%/96%	99%/92%	100%/100%	99.3%/96%
80%	97%/96%	94%/96%	100%/100%	97%/97.3%
95%	99%/96%	97%/96%	100%/100%	98.7%/97.3%

(The training set is on the left side of the ‘/’ symbol and the test set is on the right side of the ‘/’ symbol).

**Table 6:** Comparison of results before and after compensation.

Relative Humidity	SVM	KNN	ANN	Average Rate of Correct Classification
pre-compensation	92%/84%	89%/88%	97%/91%	92.7%/87.7%
post-compensation	97%/99%	97%/99%	100%/100%	98%/99.3%

(The training set is on the left side of the ‘/’ symbol and the test set is on the right side of the ‘/’ symbol).

## Data Availability

Data are contained within the article.
